# Significant enhancement of nitrous oxide energy yields from wastewater achieved by bioaugmentation with a recombinant strain of *Pseudomonas aeruginosa*

**DOI:** 10.1038/s41598-018-30326-8

**Published:** 2018-08-09

**Authors:** Ziyu Lin, Dezhi Sun, Yan Dang, Dawn E. Holmes

**Affiliations:** 1 Beijing Key Laboratory for Source Control Technology of Water Pollution, Engineering Research Center for Water Pollution Source Control and Eco-remediation, Beijing Forestry University, Beijing, 100083 China; 2 College of Environmental Science and Engineering, Beijing Forestry University, Beijing, 100083 China; 3 Department of Physical and Biological Sciences, Western New England University, 1215 Wilbraham Rd, Springfield, MA 01119 United States

## Abstract

Nitrous oxide (N_2_O) is formed during wastewater nitrogen removal processes. It is a strong greenhouse gas, however, if properly captured it can also be used as a renewable energy source. In this study, a *nosZ*-deficient strain of *Pseudomonas aeruginosa* was constructed. During growth under denitrifying conditions, the *nosZ*-deficient strain was more highly transcribing other genes from the denitrification pathway (*narG*, *nirS*, and *norB*) than the wild-type strain. This strain could also convert 85% of NO_2_^−^-N to N_2_O when it was grown with acetate compared to <0.6% by the wild-type strain. When a bioreactor treating synthetic wastewater with high NO_2_^−^-N concentrations (700 mg/L) was inoculated with this strain, the N_2_O conversion efficiencies were >73% and N_2_O comprised 73~81% of the biogas being generated. The energy yield from wastewater in bioaugmented reactors also reached levels as high as 1260 kJ/m^3^. These results are significant and show that bioaugmentation of reactors during denitrification treatment processes with *nosZ*-deficient strains of *Pseudomonas* or other core denitrifying bacteria might be an effective way to enhance N_2_O recovery.

## Introduction

Nitrous oxide (N_2_O) is a strong greenhouse gas with an atmospheric lifetime of ~120 years and a greenhouse effect that is 296 times stronger than CO_2_^1^. A significant proportion of global N_2_O emissions (3.2–10%) can be attributed to nitrogen removal processes carried out during wastewater treatment^2–6^. Although N_2_O has many negative impacts if released into the environment, recent studies have shown that N_2_O can serve as a renewable energy source^7,8^. In fact, N_2_O has a positive enthalpy of formation, and can release 82 kJ/mol when decomposed (eqn. 1). N_2_O is also a more powerful oxidant in combustion reactions than O_2_. For example, stoichiometric combustion of 1 mol CH_4_ with N_2_O releases roughly 30% more energy than stoichiometric combustion of 1 mol CH_4_ with O_2_^9^ (eqns 2–3).

1 $${{\rm{N}}}_{2}{\rm{O}}\to 1/2{{\rm{O}}}_{2}+{{\rm{N}}}_{2},\,{\rm{\Delta }}{\hat{H}}_{C}^{^\circ }=-\,82\,kJ/mol$$

2 $${{\rm{CH}}}_{4}+4{{\rm{N}}}_{2}{\rm{O}}\to {{\rm{CO}}}_{2}+2{{\rm{H}}}_{2}{\rm{O}}+4{{\rm{N}}}_{2},\,{\rm{\Delta }}{\hat{H}}_{C}^{^\circ }=-\,1219\,kJ/mol\,$$

3 $${{\rm{CH}}}_{4}+2{{\rm{O}}}_{2}\to {{\rm{CO}}}_{2}+2{{\rm{H}}}_{2}{\rm{O}},\,{\rm{\Delta }}{\hat{H}}_{C}^{^\circ }=-\,890\,kJ/mol$$

Therefore, N_2_O is commonly used to supercharge the engines of high performance vehicles (i.e. “Nitrox”) and as an oxidant in hybrid rocket motors in the aerospace industry^9^.

Researchers have started to examine the possibility of stimulating N_2_O emissions from bioreactors undergoing wastewater nitrogen removal treatment processes^4,9–12^. Many nitrogen removal treatment approaches utilize bacteria present in wastewater that can reduce nitrate or nitrite to nitrogen gas under anaerobic conditions^13^. N_2_O is an intermediate in most denitrification pathways^11^ and many researchers have tried to adjust operational parameters in bioreactors to maximize N_2_O output^12,14–19^. Unfortunately, these attempts have resulted in low conversion rates of total nitrogen to N_2_O (usually <10%), or unstable reactors^4,11,12^. It does not help that the solubility of N_2_O in pure water is extremely high at room temperature (i.e. 1500 mg/L)^20^. Therefore, when wastewater with low total nitrogen content (i.e. municipal wastewater) is treated, the majority of N_2_O is dissolved in the aqueous phase and flows out with the effluent.

One promising strategy called Coupled Aerobic–anoxic Nitrous Decomposition Operation (CANDO) utilizes a three-step process to remove nitrogen from wastewater while generating N_2_O that is then used for catalytic decomposition to N_2_ and O_2_ or as an oxidant for CH_4_ combustion^9^. This approach involves the partial nitrification of NH_4_^+^ to NO_2_^−^ which is then anaerobically reduced to N_2_O. Scherson, *et al*.^9^ found that if they supplemented reactors with pulses of electron donor (acetate) followed by pulses of electron acceptor (NO_2_^−^) during the denitrification stage of treatment, they could suppress the growth of complete denitrifiers (N_2_-producing) while slightly stimulating the growth of incomplete denitrifiers (N_2_O-producing) and polyhydroxybutyrate (PHB) accumulating denitrifiers that could couple the oxidation of PHB with nitrite reduction. This approach resulted in an N_2_O conversion efficiency of 60–65%, however, the majority of N_2_O was dissolved in the aqueous phase making energy recovery difficult.

Some denitrifying bacteria from such genera as *Pseudoxanthomonas*^21^, *Stenotrophomonas*^22^, *Luteimonas*^22^, *Corynebacterium*^23^ *and Moraxella*^24^, have a truncated denitrification pathway and their chromosomes lack the gene coding for nitrous oxide reductase (NosZ) which means that N_2_O is their terminal denitrification product. However, conditions maintained in wastewater treatment reactors are not optimal for growth of these species, and they are seldomly enriched during wastewater treatment^25,26^. It seems much more practical to focus efforts on organisms that are known to be significant members of the wastewater community during nitrogen removal treatment processes^25,26^.

**Figure 1:**
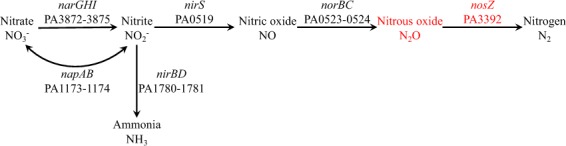
Nitrogen metabolism pathways utilized by *Pseudomonas aeruginosa*.

*Pseudomonas aeruginosa* is one example of a denitrifying bacterium that is frequently enriched in wastewater treatment plants and bioreactors^9,27,28^. Although *P*. *aeruginosa* is a complete denitrifier (nitrogen metabolism pathways of *P*. *aeruginosa* are shown in Fig. 1), it is genetically tractable and can survive when the *nosZ* gene is deleted from its chromosome^29^. Therefore, a lab-scale moving-bed biofilm reactor (MBBR) system treating high strength nitrogen synthetic wastewater was bioaugmented with a *nosZ*-deficient strain of *P*. *aeruginosa*. Significant concentrations of N_2_O were obtained in biogas generated by this system and N_2_O conversion efficiencies were higher than previously reported studies.

## Results

### Enhanced N_2_O production by *nosZ*-deficient mutant strain of *Pseudomonas aeruginosa* in pure culture studies

Nitrous oxide is formed as an intermediate by *P*. *aeruginosa* during denitrification (Fig. 1). Nitrate is first reduced by nitrate reductase (NarGHI) to nitrite, which is then reduced to nitric oxide by nitrite reductase (NirS). Nitric oxide is reduced by nitric oxide reductase (NorBC) to nitrous oxide and finally nitrous oxide is converted to nitrogen gas by nitrous-oxide reductase (NosZ). In an attempt to halt the pathway at nitrous oxide formation, *nosZ*-deletion mutant strains were constructed.

**Figure 2:**
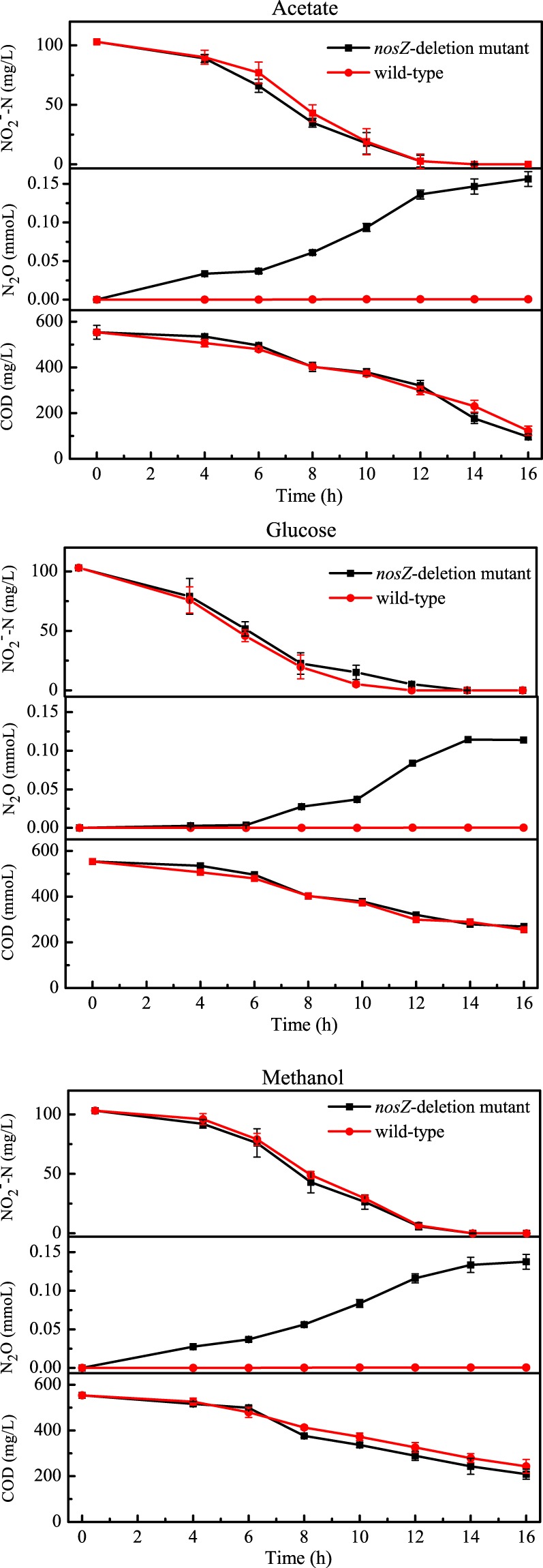
Growth of wild-type and *nosZ*-deficient mutant strains of *P*. *aeruginosa* with acetate (8.6 mM), glucose (3.0 mM), or methanol (11.5 mM) provided as electron donors and nitrite (7.2 mM) as electron acceptor. Decreases in nitrite and chemical oxygen demand (COD) concentrations and accumulation of total nitrous oxide (from liquid and gaseous phases) were monitored over time. Each point represents the average of triplicate samples.

The formation of nitrous oxide over time was measured in cultures of *nosZ*-deficient and wild-type strains with nitrite (7.2 mM) provided as the electron acceptor and either acetate (8.6 mM), glucose (3.0 mM), or methanol (11.5 mM) as electron donor (Fig. 2). Both the wild-type and *nosZ*-deficient strains grown in the presence of all three electron donors reduced almost all of the nitrite within 16 hours, demonstrating that deletion of the *nosZ* gene did not impair growth. Although nitrite reduction efficiencies were similar in both strains, significantly more nitrous oxide accumulated in the *nosZ*-deficient cultures. Almost undetectable levels (<0.0006 mmol) of N_2_O were detected in wild-type cultures, while >0.11 mmol N_2_O was detected in *nosZ*-deficient cultures grown on all three electron donors. The N_2_O conversion efficiencies of the *nosZ*-deficient mutant grown under all conditions were higher than 60% compared to <0.6% in the wild-type cultures.

**Figure 3:**
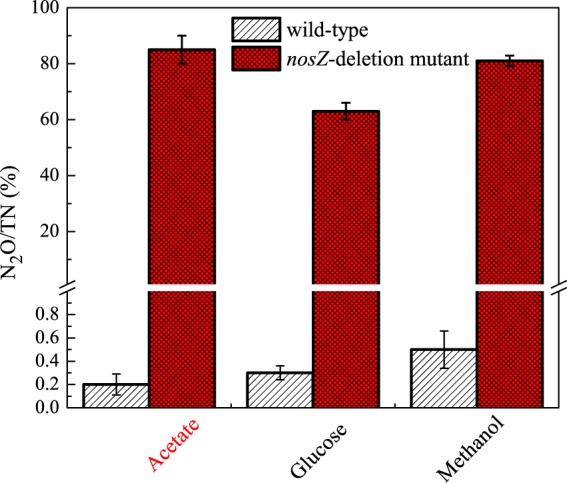
The proportion of electron acceptor (NO_2_^−^-N) that was converted to N_2_O by wild-type and *nosZ*-deficient mutant strains of *P*. *aeruginosa*. Error bars represent triplicate cultures.

When the *nosZ*-deficient mutant was grown in the presence of acetate and methanol, 81–85% of the NO_2_^−^ provided as electron acceptor was reduced to N_2_O (Fig. 3). The mutant strain only reduced 62% of the provided NO_2_^−^ to N_2_O when it was grown with glucose provided as the electron donor. It is possible that some of the electrons from glucose were being shuttled into a fermentative pathway^30,31^ which would explain the lower yield observed with this substrate. However, further investigation into this possibility is required.

### Transcription of nitrogen metabolism genes by the *nosZ*-deletion mutant strain

**Figure 4:**
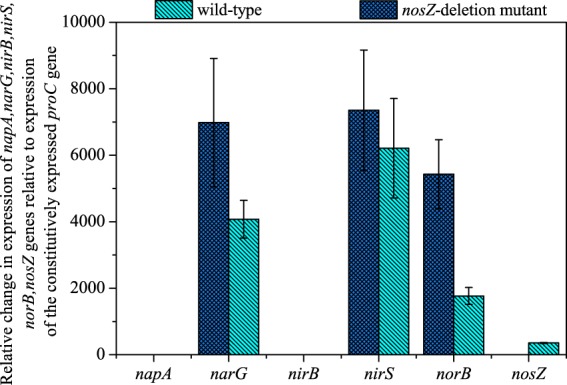
The number of *napA*, *narG*, *nirB*, *nirS*, *norB*, and *nosZ* mRNA transcripts normalized against the number of *proC* mRNA transcripts detected in wild-type and *nosZ*-deletion mutant strains of *P*. *aeruginosa* grown with acetate (8.6 mM) provided as the electron donor and nitrite (7.2 mM) as the acceptor. Error bars were calculated from triplicate samples.

Quantitative reverse transcription-PCR (qRT-PCR) was conducted with primers targeting denitrification genes (Fig. 1) in order to determine whether deletion of *nosZ* influenced transcription of other genes in the pathway. The number of mRNA transcripts from *norB*, the gene coding for the cytochrome *c* subunit of nitric oxide reductase, was 3.1-fold higher in *nosZ*-deletion strains than it was in wild-type strains (Fig. 4). Genes coding for nitrite reductase (*nirS*) and nitrate reductase (*narG*) were also more significantly expressed in *nosZ*-deficient strains. These results indicate that deletion of *nosZ* enhances transcription of genes from *nar*, *nir*, and *nor* operons. It has already been shown that the repression of transcription of *nor*, *nir*, and *nar* genes by the repressor protein Dnr is lifted by NO^32,33^. It is possible that N_2_O also helps induce transcription of nitrate, nitrite and nitrous oxide reductases as has been proposed in *Rhodobacter sphaeroides*^34^. In addition to induction of the *nar* promoter by NO, transcription of genes from the *nar* operon can also be induced by the presence of nitrate^32^. Although nitrate was never added to the cultures, low concentrations of nitrate (~0.5 mM) were abiotically formed in both the wild-type and *nosZ*-deficient cultures.

No transcripts for genes that code for periplasmic nitrate reductase (*napA*) and the large subunit from assimilatory nitrite reductase (*nirB*) were detected in either the wild-type or the *nosZ*-deletion mutant cultures. These results are consistent with previous studies that have shown that *napAB* is expressed under aerobic and microaerophilic conditions^35,36^, and that *nirB* transcription is only induced under high nitrate concentrations^37^.

### Performance of *nosZ*-deficient mutant strain in a continuous flow partial denitrification bioreactor treating synthetic wastewater

Although pure culture studies demonstrated that the *nosZ*-deficient strain generated significantly more N_2_O than the wild-type strain, it was necessary to determine 1) whether the *nosZ* deletion mutant strain would behave in a similar manner during growth in long-term continuous flow bioreactors, and 2) whether N_2_O gas generated by this strain during denitrification of high strength total nitrogen wastewater could be efficiently recovered from the bioreactor.

**Figure 5:**
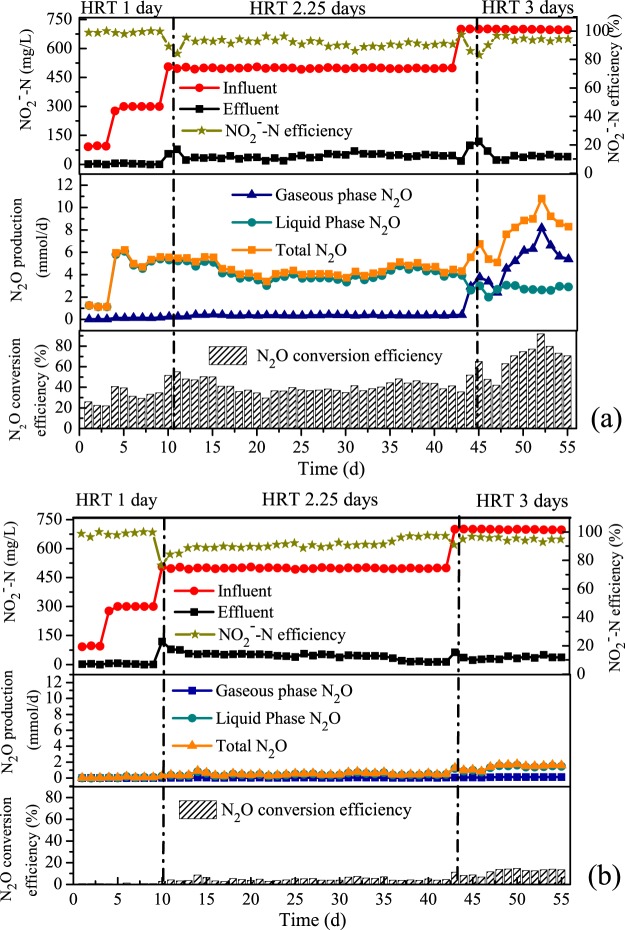
Performance of the MBBRs bioaugmented with (**a**) *nosZ*-deficient mutant or (**b**) wild type strains of *P*. *aeruginosa*.

Two denitrification MBBRs treating synthetic anaerobically-treated wastewater with nitrite as the electron acceptor and sole nitrogen source and acetate as the electron donor were operated for 55 days. One of these reactors was bio-augmented with the *nosZ*-deficient mutant strain (the experimental reactor) and the other reactor was inoculated with the wild-type strain (the control reactor). Throughout the experiment, the nitrogen loading rate was maintained at 0.2~0.3 kgN/(m^3^ d) to ensure that the denitrification working load was not too high, and the COD/N ratio was maintained at 5.0 to ensure that sufficient electron donor was available for denitrification. Nitrogen removal was >90% in both reactors (Fig. 5), indicating that they were operating stably and efficiently.

During the first stage of the experiment (Stage I: days 1–9), NO_2_^−^-N concentrations ranged from 100 to 300 mg/L and COD concentrations ranged from 500 to 1500 mg/L (7.9 to 23.8 mM acetate) (Fig. 5). Little N_2_O (0.04~0.21 mmol/d) was detected in the control reactor during Stage I. However, approximately 1.1 mmol/d and 5.5 mmol/d of N_2_O were generated by the experimental reactor when it was provided with NO_2_^−^-N concentrations of 100 mg/L and 300 mg/L, respectively. The majority (>97%, Fig. 5a) of N_2_O being generated in the experimental reactor during Stage I was associated with the dissolved liquid phase, and N_2_O gas only accounted for 3.6–5.0% of the biogas (Fig. 5). The daily N_2_O conversion efficiency from NO_2_^−^-N was 34% in the experimental reactor, nearly 50 times higher than the conversion efficiency from the control reactor (0.7%).

**Figure 6:**
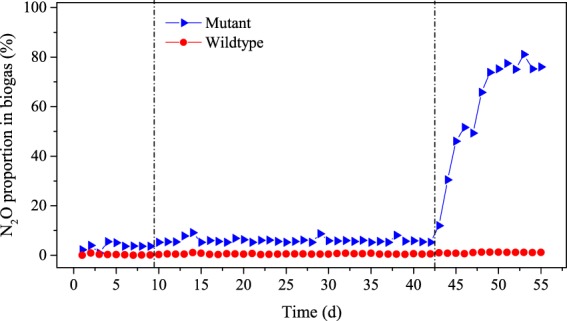
Proportion of N_2_O in biogas generated by the experimental reactor (bioaugmented with the *nosZ*-deficient mutant strain of *P*. *aeruginosa*) and the control reactor (bioaugmented with the wild-type strain of *P*. *aeruginosa*).

On day 10 (Stage II: days 10–42), NO_2_^−^-N influent concentrations were increased to 500 mg/L, COD was increased to ~2500 mg/L (39.6 mM acetate) and the hydraulic retention time (HRT) was increased from 1 day to 2.25 days. N_2_O production by the experimental reactor decreased slightly to ~4.5 mmol/d during this stage (Fig. 5) and the majority of N_2_O was still found in the liquid phase (90–92%). N_2_O accounted for a higher proportion of biogas (5.5–9.1%) than Stage I (Fig. 6), and the daily N_2_O conversion efficiency from NO_2_^−^-N increased to ~41%. In the control reactor only ~0.5 mmol N_2_O was produced per day and 4.7% of the removed NO_2_^−^-N was converted to N_2_O.

In the final stage of the experiment (Stage III: days 43–55), influent NO_2_^−^-N and COD concentrations were increased to 700 mg/L and ~3500 mg/L (55.4 mM acetate), and HRT was increased to 3 days (Fig. 5). In the experimental reactor, N_2_O production rates increased to >9 mmol/d and N_2_O accounted for 73–81% of the biogas (over 200 ml/d) being generated by the reactor. The N_2_O conversion efficiency also increased during Stage III to over 73% (Fig. 6). In the control reactor, on the other hand, N_2_O production was still low (<1.7 mmol/d) and the N_2_O conversion efficiency was <14%.

## Discussion

These results clearly showed that bioaugmentation of simple reactors with *nosZ*-deficient mutant strains of *P*. *aeruginosa* significantly improved N_2_O production. While N_2_O conversion efficiencies of *nosZ*-mutant amended reactors reached >73%, previously reported partial nitrification/denitrification reactors operated in a similar manner yielded N_2_O conversion efficiencies of only 0.8∼7.2%^19,38,39^. Previous studies controlled operational parameters to enhance N_2_O conversion efficiencies without considering the fact that much of the N_2_O was reduced to N_2_ during the final step of the denitrification pathway. However, by eliminating the final step through deletion of the *nosZ* gene, we ensured that much of the N_2_O remained in the reactor system, and this allowed us to achieve significantly higher N_2_O conversion efficiencies.

Although reactors treating synthetic wastewater with the CANDO approach had conversion efficiencies of 60–65%^9^, which were still lower than our results, this approach required supplementation of reactors with pulses of electron donor (acetate) and electron acceptor (NO_2_^−^) during the denitrification stage which is not always feasible for all wastewater treatment systems. In addition, the need to dose with only acetate during CANDO limits its treatment of actual wastewater with complex organic matter. *P*. *aeruginosa*, on the other hand, can utilize a variety of organic compounds found in wastewater as a source of electrons for denitrification^40^, and high N_2_O conversion efficiencies (62–85%) were obtained by the *nosZ*-deficient strain when it was provided with three different electron donors (acetate, methanol, and glucose).

Nitrite concentration and HRT in the experimental reactor appeared to have a significant impact on N_2_O conversion efficiencies and the proportion of N_2_O found in the biogas. For example, when influent NO_2_^−^-N was lower than 500 mg/L with an HRT of 1.0 d or 2.25 d, the N_2_O conversion efficiency was 34–41% and N_2_O only accounted for <6% of the biogas. However, when the influent NO_2_^−^-N concentration was increased to 700 mg/L with an HRT of 3.0 d, N_2_O conversion efficiencies increased to >73% and 73–81% of the biogas was composed of N_2_O. Higher nitrite concentrations resulted in a higher production of N_2_O, which became saturated in the aqueous phase and readily emitted into the gaseous phase, thereby increasing N_2_O biogas content.

The effect of HRT on N_2_O production and conversion can be explained by the fact that, similar to other denitrification bioreactors, the reactors in this study were operated under anoxic conditions. The feedstock (synthetic wastewater) contained some dissolved oxygen, which competes with nitrite as an electron acceptor and decreases N_2_O production and conversion^5^. When the HRT was short (i.e. 1.0 d), the effects of dissolved oxygen on denitrification and N_2_O production were more significant because of the relatively faster feeding speed. However, when the HRT was prolonged to 3.0 d at Stage III, dissolved oxygen was fed to the reactor at a much slower rate making conditions less oxic and more amenable to denitrification. This might also help explain the finding that N_2_O conversion rates were consistently lower in bioreactors being continuously fed anoxic wastewater than in pure culture studies carried out in anaerobic serum bottles.

**Figure 7:**
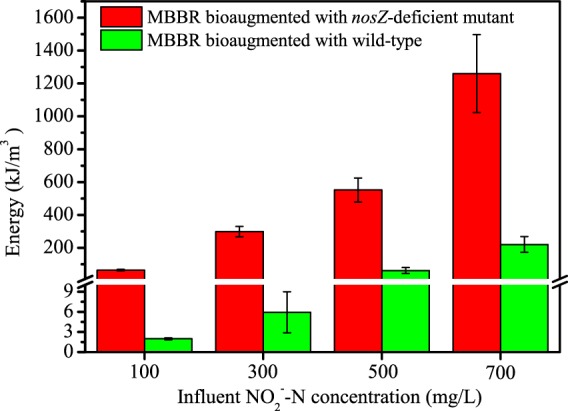
Energy recovery from N_2_O generated by the experimental reactor (bioaugmented with the *nosZ*-deficient mutant strain of *P*. *aeruginosa*) and the control reactor (bioaugmented with the wild-type strain of *P*. *aeruginosa*) when reactors were fed four different influent nitrite concentrations. Error bars represent standard deviations between operational days from each stage.

According to eqn. 1, there were significant differences in energy recovery between the experimental and control reactors at all four stages of the experiment when synthetic wastewater contained 100, 300, 500, or 700 mg/L of NO_2_^−^-N (Fig. 7). While the experimental reactor amended with the *nosZ*-deficient mutant strain recovered 64, 298, 552 and 1260 kJ/m^3^ of energy from N_2_O, only 2.0, 5.9, 61 and 220 kJ/m^3^ were recovered from the control reactor. These results showed that throughout operation, energy yields were 5.7~50 times higher in the experimental than the control reactor.

These results show great promise as a way to recover N_2_O as an energy source during nitrogen removal processes conducted on high-strength nitrogen wastewater such as leachate^41^. However, application to real-world treatment strategies needs to be evaluated in further studies.

## Methods

### Bacterial strains, plasmid and pure culture conditions

*Pseudomonas aeruginosa* PAO1 was obtained from Dr. Yihe Ge’s laboratory culture collection^42^. *Pseudomonas* cells were grown under anaerobic conditions at 37 °C in 50 ml modified AB minimal media^43^ with 8.6 mM acetate, 11.5 mM methanol or 3.0 mM glucose as electron donor (to theoretically provide the equal COD and electrons) and 7.2 mM NO_2_^−^-N (100 mg/L-N) as the electron acceptor for denitrification. All 50 ml cultures were inoculated with 1 ml of *P*. *aeruginosa* cells; ~2.5 × 10^8^ cells which had a biomass of 2.54 mg/ml.

*Escherichia coli* DH-5α and *E*. *coli* S17.1 cells were grown in LB media at 37 °C. If appropriate, gentamycin was added at final concentrations of 15 µg/ml for *E*. *coli* and 30 µg/ml for *P*. *aeruginosa*. The suicide plasmid used in this study was pEXG2^44^.

### Construction of *nosZ*-deletion mutant of *Pseudomonas aeruginosa* PAO1

The sequences of all primer pairs used for construction of the *nosZ* deletion mutant are listed in Supplementary Table S1. Primer pairs were designed to amplify regions approximately 500 bp upstream and downstream of the *nosZ* gene in *P*. *aeruginosa* PAO1. PCR was used to add BamHI (GGATCC) and AvrII (CCTAGG) restriction sites to the 5′ and 3′ regions of the *nosZ* upstream fragment and HindIII (AAGCTT) and AvrII to the 5′ and 3′ regions of the *nosZ* downstream fragment. PCR products were digested with their appropriate restriction endonucleases (NEB, Beverly, MA), and ligated into the suicide vector pEXG2, resulting in the formation of pEXG2up5′ + 3′dn. Sanger sequencing was done to verify the sequence of the cloned product. A gentamycin resistance cassette was amplified via PCR with flanking AvrII restriction sites using the pBSL141 plasmid as a template. After digestion of both the gentamycin cassette and the pEXG2up5′ + 3′dn plasmid with AvrII, the gentamycin cassette was ligated into the pEXG2up5′ + 3′dn plasmid.

Following plasmid construction, the pEXG2up5′+ gentamycin cassette +3′dn plasmid was chemically transformed into *E*. *coli* S17.1 cells and grown on LB agar plates supplemented with 15 µg/ml gentamycin.

Conjugation was then used to transfer the recombinant pEXG2 plasmid into *P*. *aeruginosa* PAO1 according to previously described methods^45^. Deletion of the *nosZ* gene from the *P*. *aeruginosa* chromosome was verified by sequencing (see Supplementary Figs S1, S2).

### RNA extraction

RNA was prepared from triplicate cultures of the *P*. *aeruginosa* PAO1 *nosZ*-deletion mutant and wild-type strains grown in modified AB minimal media with 8.6 mM acetate as the electron donor and 7.2 mM NO_2_^−^ as the electron acceptor. All solutions used during the RNA extraction process were prepared with DEPC-treated water (Adil). Cells were split into 50-ml conical tubes (BD Biosciences, San Jose, CA) and pelleted by centrifugation at 3,000 *g* for 15 min. RNA was then extracted with the RNAprep Bacteria kit (TianGen, China) and treated with DNA-free DNase (Ambion) according to the manufacturer’s instructions. All RNA samples were checked for integrity by agarose gel electrophoresis and had *A*_260_/*A*_280_ ratios of 1.8 to 2.2. In order to ensure that RNA samples were not contaminated with DNA, PCR amplification with primers targeting the 16S rRNA gene was conducted on RNA samples that had not undergone reverse transcription.

### Quantitative RT-PCR analysis of denitrification pathway genes

Complementary DNA (cDNA) was generated from total RNA (1 μg) extracted from *P*. *aeruginosa* PAO1 cells with the FastQuant reverse transcriptase Kit (TianGen, China) according to the manufacturer’s instructions. This cDNA was then used as a template for various PCRs. Sequence data obtained from the DOE Joint Genome Institute (JGI) website (www.jgi.doe.gov) were used to design qRT-PCR primers. All qRT-PCR primers (See Supplementary Table S1) were designed according to the manufacturer’s specifications (amplicon sizes of 100 to 200 bp), and representative products from each of these primer sets were verified by sequencing. qRT-PCR amplification and detection were performed with the 7500 real-time PCR system (Applied Biosystems). Optimal qRT-PCR conditions were determined using the manufacturer’s guidelines. Each PCR mixture consisted of a total volume of 25 µL and contained 1.5 µL of the appropriate primers (stock concentrations, 15 µM) and 12.5 µL SYBR green PCR master mix (Bio-Rad).

### Lab-scale denitrification bioreactor set-up

Two 1.5 L lab-scale anaerobic MBBRs (Φ8.0 cm × 30.0 cm) were set up to carry out the partial denitrification treatment of synthetic wastewater with acetate as the electron donor and nitrite as the electron acceptor. NO_2_^−^-N concentrations were stepwise increased from 100 mg/L to 700 mg/L, and the COD/N ratio was maintained at 5.0 for the entire period of operation. Each liter of synthetic wastewater also contained 1.0 g K_2_HPO_4_, 0.025 g CaCl_2_, 0.0125 g MgCl_2_ and 1 mL of trace element solution^9^. The pH of the synthetic wastewater was adjusted to 8.0 to avoid inhibition of denitrification caused by free nitrous acid^46^.

One reactor (the experimental reactor) was inoculated with the *nosZ*-deficient mutant strain of *P*. *aeruginosa*, and the other reactor (the control reactor) was inoculated with the wild-type strain of *P*. *aeruginosa* PAO1. Both reactors were filled with high-density polyethylene bio-carriers (60% vol). The bio-carriers provided a large surface area for microorganism colonization (Φ7.0 mm × 9.0 mm, specific surface area 900 m^2^/m^3^). Two reactors were operated at 37 ± 1 °C during the entire period, and the HRT was stepwise increased from 1.0 day to 3.0 days as the nitrogen loading increased to maintain the nitrogen loading rate at 0.2~0.3 kgN/(m^3^ d). The biomass concentration in the bioreactor was in the range of 0.7–2.1 gMLVSS/L. The influent/effluent and biogas were sampled every 24 h.

### Analytical methods

COD, NO_2_^−^-N, NO_3_^−^-N and NH_4_^+^-N were determined by standard methods^47^. Cell growth was determined with a spectrophotometer (UV-1800, Shimadzu, Japan) set at a wavelength of 600 nm (OD600). pH was measured with a HACH sensION+ pH3® pH meter (HACH, USA). Oxidation–reduction potential (ORP) was measured with an ORP Meter (M 300, Mettler Toledo, Greifensee, Switzerland) equipped with a Redox Electrode. The ORP of the reactors were maintained at <0 mV throughout the operational periods. Organic acids were measured with high-performance liquid chromatography (HPLC) as previously described^48^. Total biogas volume in the 2 L-gas-sampling bag was monitored every 24 h with a digital mass flow meter (FMA4000; Omega; USA). The N_2_O portion of biogas was analyzed with a gas chromatograph (Agilent 7890 A) equipped with an electron capture detector (ECD). N_2_O dissolved in the liquid phase was calculated with Henrys law as previously described^49^. Statistical differences were determined by one-way analysis of variance (ANOVA) with *p* < 0.05 considered to be statistically significant.

### Data availability

All data generated or analysed during this study are included in this published article (and its supplementary information files) and are available from the corresponding author on reasonable request.

## Electronic supplementary material

Supplementary material
