# Supplementary material for: Significant enhancement of nitrous oxide energy yields from wastewater achieved by bioaugmentation with a recombinant strain of Pseudomonas aeruginosa
Source: Sci Rep. 2018 Aug 9;8:11916. doi: 10.1038/s41598-018-30326-8 (PMC6085377; doi:10.1038/s41598-018-30326-8)
Supplement: Supplementary file 1 — Supplementary material [file 41598_2018_30326_MOESM1_ESM.docx]

**Significant enhancement of nitrous oxide energy yields from wastewater achieved by bioaugmentation with a recombinant strain of *Pseudomonas aeruginosa***

Ziyu Lin^a,b^, Dezhi Sun^a^*, Yan Dang^b^*, Dawn E Holmes^c^

**Supplementary Table S1. Primer sequences used in this study**

| Construction of *nosZ* deletion mutant strain | |
| --- | --- |
| Primers | Primer sequence |
| PA_nosZ_Up_Fwd | TATGGATCC ATCAAGTACATCATCCTGCTGGT |
| PA_nosZ_Up_Rev | TATCCTAGG CATCTCGTGTCGTCCTCAAG |
| PA_nosZ_Dwn_Fwd | TATCCTAGG CACGCCCTGCACATGGAAATGT |
| PA_nosZ_Dwn_Rev | TATAAGCTT GTCCTTCACCGCATAGAGGT |
| Gent_Fwd | CCTAGGTCGAATTGACATAAGCCTGTTC |
| Gent_Rev | CCTAGGACGAATTGTTAGGTGGCGGTA |
| qRT-PCR of denitrification genes | |
| Q-RT-PCR Primers | Primer sequence |
| napA_1319f | CGGAGAAGATCTGGAAGGTG |
| napA_1449r | CTGCATGTTGTTGCTGACCT |
| narG_1828f | AACGGCACCAGCTTCTTCTA |
| narG_1950r | GTTGTAGTCCAGGGCGTGTT |
| nirS_1293f | GCAGTACGCCTGGAAGAAAG |
| nirS_1395r | GGTGGTGTCGACGTAGAGGT |
| norB_567f | CTACAACCCGGAAAACCTCA |
| norB_688r | TGATCTTCACCAGCACGAAG |
| nosZ_1144f | GCATACCCGCTACGTGTTCT |
| nosZ_1243r | GTACAGGGTGAAGGCGTTGT |
| nirB_53f | CCCTGGAAGAACTGCTCAAG |
| nirB_188r | AGGACGATTTCCTCGAAGGT |
| proC_649f | CTGTCCAGCGAGGTCGAG |
| proC_822r | TTATTGGCCAAGCTGTTCG |

(A)
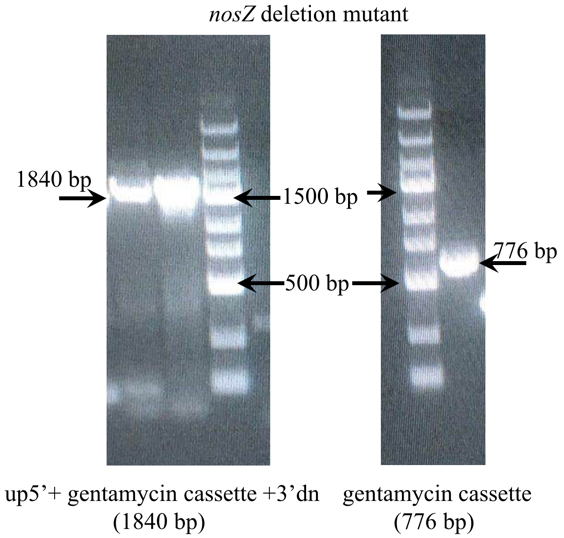
 (B) **
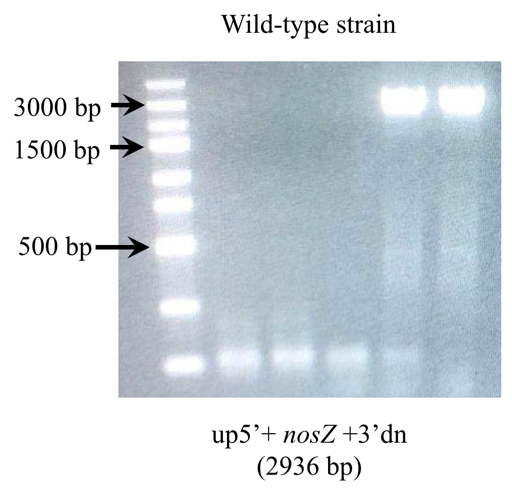
**

**Supplementary Figure S1. PCR product of *nosZ* region including 500 bp upstream and downstream of the gene using PA_nosZ_Up_Fwd and PA_nosZ_Dwn_Rev. The gentamycin cassette was also amplified from the mutant strain with Gent_Fwd and Gent_Rev.** The full-length gels of this figure are place below.

The full-length gel photo of Figure S1(A):


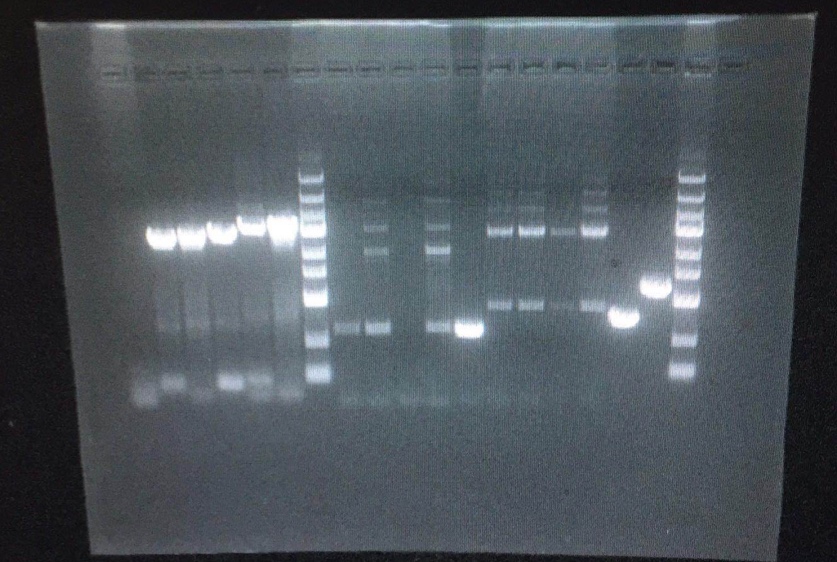


The full-length gel photo of Figure S1(B):


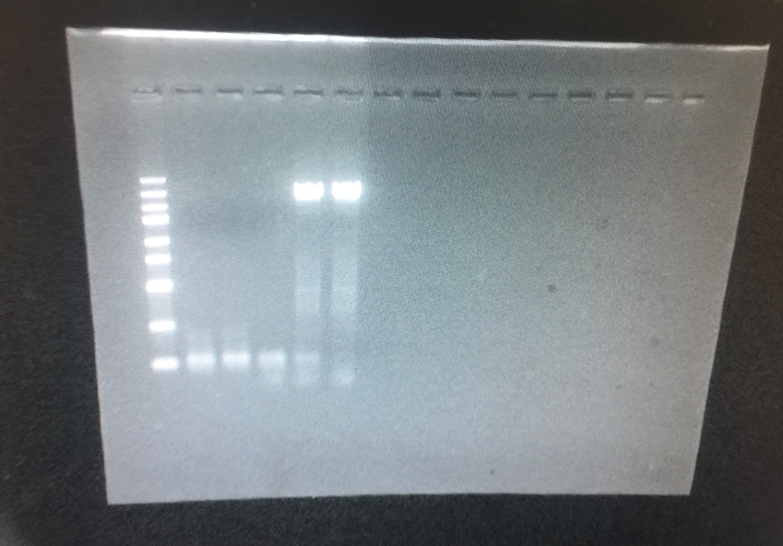


Note:

upstream region was 525 bp;

downstream region was 539 bp;

gentamycin cassette was 776 bp (Figure S1A right);

up5’+ gentamycin cassette +3’dn 1840 bp (Figure S1A left);

up5’+ *nosZ* +3’dn 2936 bp (Figure S1B).

The size of the PCR product using upstream forward and downstream reverse in the mutant was 1840 bp, while the wild-type product was 2936 bp. These results together with the detection of the gentamycin cassette in the mutant, showed that the *nosZ* gene was successfully replaced by the gentamycin cassette.

**
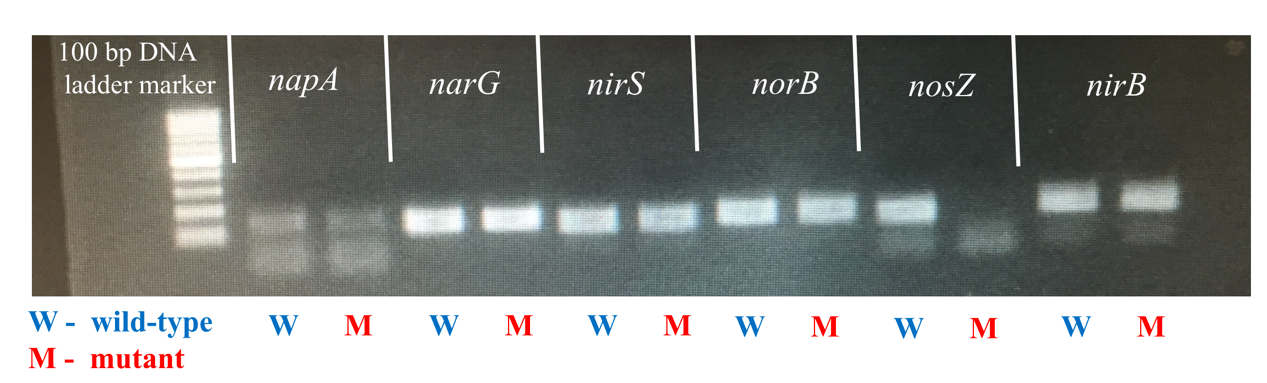
**

**Supplementary Figure S2. PCR check of denitrification pathway genes with DNA extracted from wild-type (W) and *nosZ*-deficient mutant (M) strains of *P. aeruginosa* as templates. The primers designed for qRT-PCR in Table S1 were used. These bands were from the same gel.** The full-length gels of this figure are place below.

Note: *nosZ* gene cannot be detected from the *nosZ*-deficient mutant.

The full-length gel photo of Figure S2:


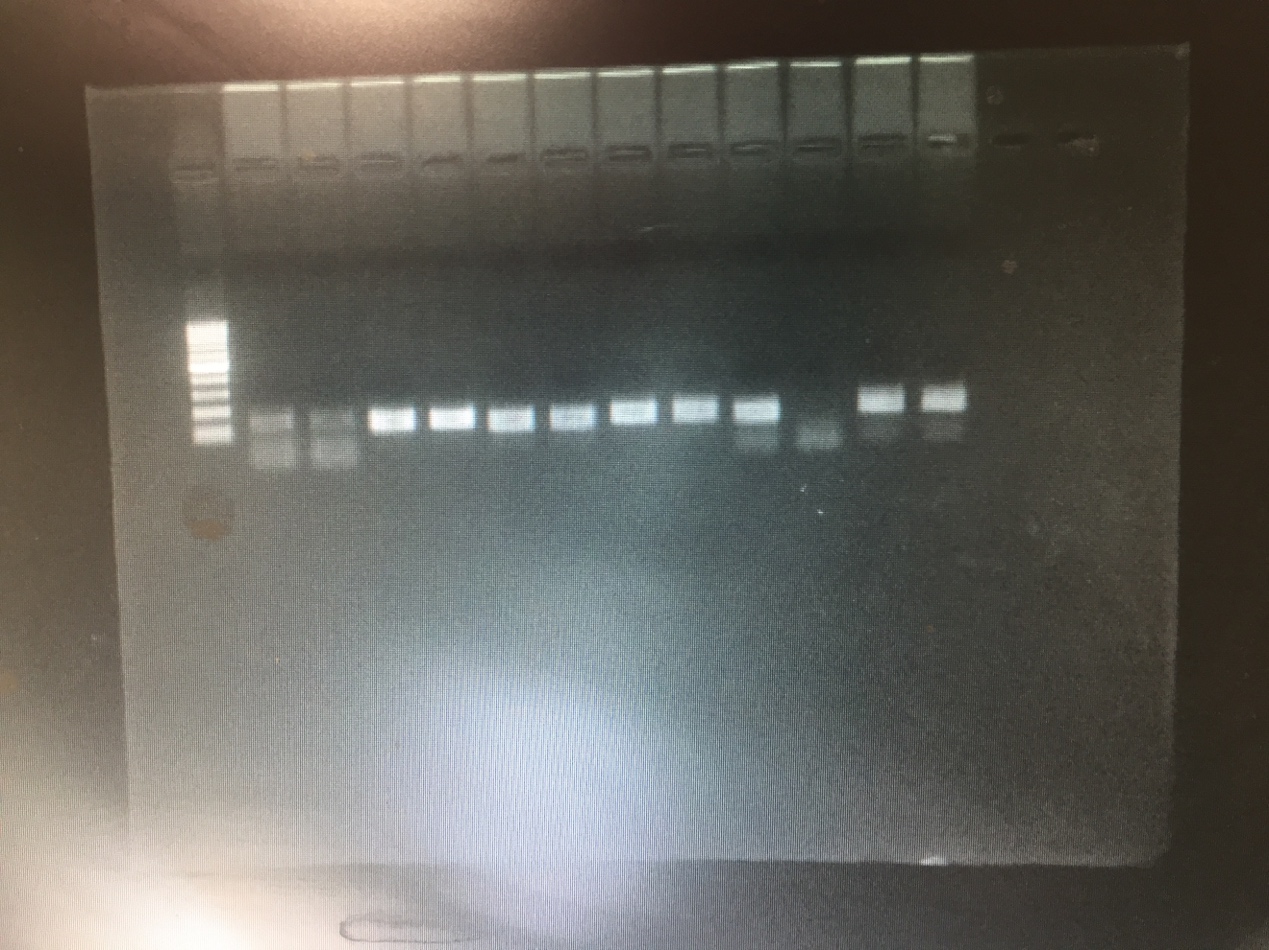


**Supplementary sequencing information: Sequencing results of *nosZ*-deficient mutant strains of *P. aeruginosa* using primer pairs of PA_nosZ_Up_Fwd and PA_nosZ_Dwn_Rev**

ATCAAGTACATCATCCTGCTGGTGCTCTTCGGTCTCTCCCTGGAATCCCTGGCGCTGGCCGAGCAGGCCGCGGAGGTGGAGCCGTTCAAGACCGCCATCACCCTCGGCTTCGACCGCCAGTGGTGGTTCGTCGCCTACGCCGTCGCGCTGCTGGTGGTCAACCTGTTCACCCGCAAGGTCTATTGCCGCTACCTCTGCCCGCTGGGCGCGGCCCTGGCGATCCCGGCCAAGGCGCGCCTGTTCGACTGGCTCAAGCGCCGTGCGGAATGCGGCAGGCCCTGCCAGCTCTGTGCCCGCGAATGCGAGATCCAGGCGATCCATCCCGACGGCCGCATCGAGGCCAACGAATGCCACTACTGCCTCGACTGCCAGATGACCTACCACGACCAGGACAAGTGCCCGCCGCTGGTGAACAAGCGCAAGAAGCGCGCGAAGAGCGCGCCGGCGGACAACGCGCGGATACCCGCGGAGAACCTCTGACCTGGCTCCCTTTCCCCTGACTGCCCTTGAGGACGACACGAGATG cctagg TCGAATTGACATAAGCCTGTTCGGTTCGTAAACTGTAATGCAAGTAGCGTATGCGCTCACGCAACTGGTCCAGAACCTTGACCGAACGCAGCGGTGGTAACGGCGCAGTGGCGGTTTTCATGGCTTGTTATGACTGTTTTTTTGTACAGTCTATGCCTCGGGCATCCAAGCAGCAAGCGCGTTACGCCGTGGGTCGATGTTTGATGTTATGGAGCAGCAACGATGTTACGCAGCAGCAACGATGTTACGCAGCAGGGCAGTCGCCCTAAAACAAAGTTAGGTGGCTCAAGTATGGGCATCATTCGCACATGTAGGCTCGGCCCTGACCAAGTCAAATCCATGCGGGCTGCTCTTGATCTTTTCGGTCGTGAGTTCGGAGACGTAGCCACCTACTCCCAACATCAGCCGGACTCCGATTACCTCGGGAACTTGCTCCGTAGTAAGACATTCATCGCGCTTGCTGCCTTCGACCAAGAAGCGGTTGTTGGCGCTCTCGCGGCTTACGTTCTGCCCAGGTTTGAGCAGCCGCGTAGTGAGATCTATATCTATGATCTCGCAGTCTCCGGCGAGCACCGGAGGCAGGGCATTGCCACCGCGCTCATCAATCTCCTCAAGCATGAGGCCAACGCGCTTGGTGCTTATGTGATCTACGTGCAAGCAGATTACGGTGACGATCCCGCAGTGGCTCTCTATACAAAGTTGGGCATACGGGAAGAAGTGATGCACTTTGATATCGACCCAAGTACCGCCACCTAACAATTCGT cctagg GAAATGTGCGGGCGGATGCTGGTGGAAAAGGCTTGATCCACGCAGCGCGGTCGCTGGCCGGGAGCCGGGCGGGGCCGCTCCTCGCCCTGCTCCTGCTCGGCCTCGCCACGGCCCGCGCGGAGCCGGTCGACGGCCTGCCGCTGCGGGCCGACGGCGATGGCCGCTGGAGCCTGGCGGCGGGCCGCTACGCCGGCAACTTCGTCATCGACCGGCCGCTGCACCTACGCTGCGAGGCCGGCGCCGAACTGGACGGCGGCGGCCACGGCAGTCTGCTGACCCTGACCAGCCCCGGGATCACCGTCGAGGGCTGCCGGCTGCGCAACTGGGGGCGCAACCTGACCGAACTCGACGCCGCGATCTTCGTCGGCAAGGCCGCCAGCGGCGCCGTGATCCGCGGCAACGACCTGCGCGGCGCGGGATTCGGCGTCTGGCTCGACGCCACGGTGGGCGCGCAGGTGCTCGACAACCGCATCGAGGGCGACGAAAGCGTGCGCTCCCAGGATCGCGGCAACGGTATCCACCTCTATGCGGTGAAGGAC

PA_nosZ_Dwn_Fwd

AvrII

PA_nosZ_Up_Rev

**gentamycin cassette**

AvrII

PA_nosZ_Up_Fwd

PA_nosZ_Dwn_Rev

Note: Sequencing result also demonstrated that the *nosZ* gene was replaced by gentamycin cassette in mutant strain of *P. aeruginosa*.
